# Self-reported physical activity status among adolescents in Debre Birhan town, Ethiopia: Cross-sectional study

**DOI:** 10.1371/journal.pone.0229522

**Published:** 2020-02-21

**Authors:** Osman Yimer Mohammed, Esubalew Tesfahun, Abdurahman Mohammed Ahmed, Alebachew Demelash Bayleyegn

**Affiliations:** 1 Department of Midwifery, College of Health Science, Debre Birhan University, Debre Birhan, Amhara Regional state, Ethiopia; 2 Department of Public Health, College of Health Science, Debre Birhan University, Debre Birhan, Amhara Regional state, Ethiopia; 3 Department of Nursing, College of Health Science, Debre Birhan University, Debre Birhan, Amhara Regional state, Ethiopia; Addis Ababa University School of Public Health, ETHIOPIA

## Abstract

**Background:**

Regular physical activity reduces the risk of ischaemic heart disease, stroke, diabetes, and breast and colon cancer. But, adolescents are insufficiently physically active. Therefore, this study was aimed to assess self- reported physical activity status and associated factors among adolescents in Debre Birhan town, Ethiopia.

**Methods:**

School based cross-sectional study was conducted from April 20 to May 10/2019 in Debre Berhan town Secondary schools. Multi-stage sampling technique was used to select 580 study participants from three secondary schools. Physical activity was assessed using questions adopted from recreation, sport, and leisure-time physical activity assessment section of international physical activity questionnaire (IPAQ). Adolescents who have done moderate to vigorous exercise for 60 minutes per day for at least three days in the last seven days were categorized as physically active. Descriptive statistics, bivariable and multivariable logistic regression analysis was done. Probability value less than 0.05 was used as a cut of point to determined statistically significant association.

**Result:**

A total of 580 students participated in this study. Less than 1 in 5 (17.2%, 95% confidence interval (CI) = 14.13%-20.27%) students were physically active. Male student (Adjusted odds ratio (AOR): 2.63, 95%CI = 1.5–4.59), age less than or equal to 16 (AOR: 2.04, 95% CI = 1.12–3.71) and access to sports center (gymnasium) (AOR: 2.09, 95% CI = 1.12–3.89 were positively associated with physical activity.

**Conclusion:**

Physical activity status was very low. Therefore, the local, regional and national administrators in collaboration with sport and health offices administrators should make facilities accessible.

## Introduction

Physical activity is defined as bodily movement due to contraction of skeletal muscles. It includes exercise, sport, active play and walking [1, 2]. The American Heart Association recommends 30–60 minutes of aerobic exercise three to four times per week, while WHO recommends on a daily basis [3, 4].

Physical activity has a protective effect against non-communicable diseases (NCD) like cardiovascular diseases, stroke, type 2 diabetes, and colon and breast cancer [5, 6]. However, 81% of adolescents are insufficiently physically active and this contributes to 3.2 million deaths each year [7]. Non-communicable diseases claimed the life of 2.7 million and 3.1 million people in the year 2012 and 2015 respectively globally [8, 9]. In Ethiopia alone, non-communicable diseases claimed 34% of all deaths in the year 2008 and rise to 40% in the year 2014 [10]. It is reported that if inactivity is decreased by 25%, more than 1.3 million deaths could be averted every year [6].

The level of physical activity varies across the globe. It is reported to be 37.1% in Sub-Saharan countries, 34.5% in the Caribbean, 19.1% near East and Central Asia, 19.1% and 26.8% in South Asia and China 26.8% and 28.1% in Scotland [11, 12].

Physical inactivity is associated with sedentary work and living environments, low economic status and disability [13]. Different quantitative investigations globally identified factors affecting physical activity like age, sex, education, maternal education, and cumulative family income level [11, 12, 14–18]. Factors like interest, time, convenience, lack of awareness, family influence, poor social support, gender and culture, availability of facilities are qualitatively identified barriers of physical activity [19–24]. On the contrary, improving fitness, wish to stay in shape, health, supportive environment, peer pressure and access to safe play area are facilitators of physical activity [21–24]. While the impact of physical inactivity and non-communicable diseases is increasing from time to time, the attempt to explore the stumbling block for active lifestyle are not well studied in Ethiopia generally and in the study area specifically. Gross school attendance ratio is 91% and adolescents, whose physical inactivity is higher, contribute the major share in the urban area [7, 25]. Therefore, this study was aimed to assess the status of physical activity and associated factors among adolescents of Debre Berhan town.

## Methods and materials

### Study design and participants

A school based cross-sectional study among secondary school adolescents was conducted in Debre Berhan town, Ethiopia from April 20 to May 10, 2019. Debre Berhan town is the capital of North Shoa administrative zone in Amhara regional state. The town is located 695 km far from Bahir Dar, the capital city of Amhara regional state and 130 km far from Addis Ababa, the capital city of Ethiopia. In 2018/19 academic year, there were 11,111 students in five secondary Schools in Debre Berhan Town.

The sample size was calculated using single population proportion formula with the following assumption; 95% confidence level, margin of error 0.05, proportion of the students who were physically active 37.1% [11], design effect 1.5 and 10% none response rate. Based on this the calculate sample size was 591.

Multi-stage sampling was used to select three secondary schools from four governmental and one private secondary Schools. Primarily the schools stratified into public and private with the assumption as there is difference in levels of physical activity and two public and one private school was selected using simple random sampling (stage1). Then a sample of 591 were allocated proportionally to total number of students in each of the secondary school students and their grade level (grade 9, 10, 11 and 12). Finally, students were selected using computer generated simple random sampling by excel spread sheet from each section of selected schools. (stage2).

### Participants/Population

In this study, the adolescent implies a segment of the population whose age fall in the age range between thirteen and nineteen years. Secondary school refers to those students who were attending their education in the grade ranges between 9 up to 12.

### Measurement

Questionnaire which addresses the dependent and the independent variables was developed. The primary outcome of this research, physical activity status, was assessed using questions adopted from recreation, sport, and leisure-time physical activity assessment section of international physical activity questionnaire (IPAQ) [14]. “Physical activity includes any moderate to vigorous physical activities done during recreation, sport, and leisure-time physical activity (aerobics, running, weight lifting, marshal art, gymnastic or fast bicycling, playing football, volley ball, basketball, walking).” Adolescents were asked how many days they were physically active and for how long during the last seven days. Then, adolescents who reported to have done moderate to vigorous exercises for 60 minutes per day for at least three days in the last seven days were categorized as physically active.

The independent variables include adolescents’ and their parents’ Socio-demographic characteristics like educational status, occupation, religion, family income, residence, age, sex and grade level. Adolescent’s perception towards regular physical activity in relation to its health benefits was also assessed. Moreover, availability of playground and gymnasium was assessed to see its effect on the status of physical activity of adolescents.

### Data quality management

The English version of the structured questionnaire was translated into the local language, Amharic and back translated to English by professional language translators and its consistency was checked by the investigators. Pretest was done on 5% the sample size among adolescents, at secondary school in the nearest town to the study site. Some items were modified after the pretest. The collected data was checked for completeness, accuracy, consistency and clarity. Training was given to data collectors and supervisors about the tool and data collection procedures and ethical issues.

### Data processing and analysis

Manually checked data, completeness and consistencies, was entered to Epidata version 4.4.2.1 software and exported to Statistical Package for Social Sciences (SPSS) version 20 software for analysis. Then the data was presented by using frequency distributions, graphs and numerical summary measures. To identify determinant factors, both bivariable and multivariable logistic regression analysis was done. Variables in the bivariable analysis with a P-value less than 0.2 were included in multivariable logistic regression analysis to control the confounding. Strength of association was measured using odds ratio with 95% confidence intervals. Statistical significance was declared at P-value less than 0.05. The model fitness was checked using Hosmer and Lemeshow goodness of fit test.

### Ethical consideration

Primarily, ethical approval was obtained from Debre Berhan University, College of Health Science research ethical approval committee. Then assent was obtained from each school administrators (guardians of the school children). Written consent was also secured from the study participants. All participants were informed their right to refuse participating in the study at any time and ask any questions related with the study. Furthermore, the data was collected anonymously and confidentiality of the data was kept throughout the investigation.

## Results

### Socio-demographic characteristics of adolescents and their parents

A total of 580 adolescents were participated in the current study, which yields a response rate of 98.14%. Among the study participating adolescents 323 (55.7%) were female, 341 (58.8%) were from grade 9 and 10 with mean age 17.32 (±1.23 SD). About 362 (62.4%) parents live in urban, 215 (37.1%) mothers and 256 (44.1%) fathers were attended secondary or higher level of education. About 239 (41.2%) of mothers were an employee or involve in income generating activities for the family and 216 (37.2%) fathers were farmers. Two hundred ninety-nine (51%) of the family had more than 4800 Ethiopian Birr of average monthly income (Table 1).

**Table 1:** Socio-demographic characteristics of adolescents and their parents in Debre Berhan town, Ethiopia, 2019.

Variables	Category	Number	Percent (%)
**Sex of adolescents**	Male	257	44.3
	Female	323	55.7
**Age of adolescents**	14–16	160	27.6
	17–19	420	72.4
**Grade**	9–10	341	58.8
	11–12	239	41.2
**Mother Education**	No formal Education	184	31.7
	Elementary	188	31.2
	Secondary and above	215	37.1
**Father Education**	No formal Education	144	24.8
	Elementary	180	31
	Secondary and above	256	44.1
**Mother Occupation**	House wife	341	58.8
	Employee	239	41.2
**Father Occupation**	Farmer	216	37.2
	Private	169	29.1
	Government and Non-governmental organization	195	33.6
**Family Monthly Income in quartiles**	≤2000	158	27.3
	2001–5000	126	21.7
	5001–7999	152	26.2
	≥8000	144	24.8
**Family Residence**	Urban	362	62.4
	Rural	218	37.6

### Adolescents access for playground and their perception towards physical activities

Of adolescents who participated in the study 339 (58.4%) had access for playground and 106 (27.6%) had also access for gymnasium around their residence. A total of 382 (65.9%) adolescents perceived that regular physical activity is very enjoyable and 575 (99.1%) perceived that it is beneficial for health (Table 2).

**Table 2:** High school adolescent’s access to playground and perceptions towards physical activities in Debre Berhan Town, Ethiopia, 2019.

Variables	Category	Number	Percent (%)
**Play Ground access**	Yes	339	58.4
	No	241	41.6
**Gymnasium access**	Yes	106	27.6
	No	474	72.4
**Regular Physical activities**	Very Enjoyable	382	65.9
**`**	Enjoyable	174	30.0
	Not Enjoyable	24	4.1
**Health Benefits of regular physical activity**	Healthy	575	99.1
	No Benefit	5	0.9
	Not healthy	0	0
**Health Impacts of Sedentary behavior**	Not Healthy	550	94.8
	No Harm	17	2.9
	Good for Health	13	2.2

### Adolescents Physical activity status

From all participants in the study, 100 (17.2%) (95%CI: 14.13–20.27) of whom [24.5% male and 11.5% female] were physically active.

### Factors associated with physical activity

In the bivariable analysis, the association between socio demographic characteristics of adolescent and their parents, media access and characteristics of the living environment, and the status of physical activity among the adolescents were assessed. All the variables which gave P-value less than 0.2 were included in the multivariable regression analysis.

Under multivariable logistic regression sex, age and availability of gymnasium around adolescents’ residence were turned to be significantly associated factors with being physically active. Accordingly, the odds of male were 2.63 (AOR 2.63, 95% CI = 1.5–4.59) times higher than their female counter parts. Similarly, the odds of being physically active for adolescents below the age of 16 years of age were 2.04 (AOR 2.04, 95% CI = 1.12–3.71) times higher than adolescents who were older than the age of 16. The odds of being physically active among adolescents who had access to gymnasium were 2.09 (AOR 2.09, 95% CI = 1.12–3.89) times higher than counter parts (Table 3).

**Table 3:** Factors associated with leisure time physical activity of adolescents among High school students in Debre Birhan, Ethiopia, 2019.

Variables		Physically		COR (95%-CI)	AOR (95%-CI)
		Active	inactive		
Sex	Male	63	194	2.51 (1.61–3.92)	2.63(1.5–4.59)
	Female	37	286	1	1
Age	≤16	37	123	1.71 (1.08–2.69)	2.04(1.12–3.71)
	>16	63	357	1	1
Mother Education	No education	20	164	1	1
	Primary	36	145	2.04 (1.13–3.68)	1.27(0.51–3.13)
	Secondary	13	75	1.42 (0.67–3.01)	0.79(0.29–2.09)
	Univ/college	31	96	2.65 (1.43–4.90)	1.867(0.59–5.85)
Father Education	No education	17	127	1	
	Primary	28	152	1.38 (0.72–2.63)	1.02(0.43–2.41)
	Secondary	19	72	1.97(0.96–4.03)	1.21(0.45–3.26)
	Univ/college	36	129	2.09 (1.12–3.90)	1.83(0.54–6.18)
Access to Television	Yes	67	278	1.48 (0.94–2.32)	0.85(0.42–1.69)
	No	33	202	1	1
Access to Mobile	Yes	55	238	1.46 (0.83–2.57)	0.85(0.39–1.87)
	No	19	120	1	1
Internet					
Using Social	Yes	21	202	1.43 (0.93–2.21)	1.38(0.67–2.84)
	No	49	278	1	1
Media					
Access to	Yes	36	70	2.28 (1.39–3.73)	2.09(1.12–3.89)
	No	97	377	1	1
Gymnasium					

checked using Hosmer and Lemeshow goodness of fit test (p-value = 0.69) and multicollinearity effect was also checked.

## Discussion

This study tried to measure the magnitude and associated factors of physical activity among adolescents in Debre Berhan town. The status of regular physical activity among high school adolescents was 17.2%. This finding is consistent with study finding in Brazil which reported that the overall physical activity was 19.69% [12]. This similarity might be due to the similarity in the socio-demographic status between the two populations. In the contrary, this result was lower compared with studies in Scotland which was 28.1 [7]. This discrepancy might be due to lack of access for playground and gymnasium. It is also lower compared with 23 low- and middle-income countries in the Sub-Sahara, which was 37.1%. The difference might be the presence of middle-income countries in the study which are different from our setup [6].

Among the total participants on the current study, around 65.9% feel regular physical activity is very enjoyable, 99.1% feel regular physical activity is healthy and 94.8% feel sedentary behavior is not healthy. Furthermore, 2.9% and 2.2% feel siting too much has no harm and good for health respectively. This result is higher in all aspect compared with study in Scotland which reported about 66% of participants consider regular exercise to be healthy but only 20–30% found exercise easy, relaxing and enjoyable [7]. The variation in the life style might be due to the difference in the level of development and life style.

Physical activity was predicted by sex; males were more likely to be involved in regular physical activity. The finding was consistent with study findings in Scotland, Czech Republic, Ghana, UK, South Africa, England and Poland [7, 9, 10, 13, 26–28]. This might be due to lack of female friendly facilities and gender role variation.

This study also identified that physical activity was determined by age; young adolescents are more likely to be involved in regular physical activity. This is in line with the study findings in 23 low and middle-income countries in Sub Sahara, Scotland, South Africa and Poland [6, 7, 9 and 13]. This could be as the age increase so does the responsibility in the family and schools to consumes most leisure times. In addition, access to gymnasium was significantly associated with physical activity. This indicates that accessibility of sport infrastructures like gymnasium around the residence helps to adopt active lifestyle.

Socio-economic related variables were not significantly associated with status of physical activity. This is not in line with the study findings in 23 low and middle- and middle-income countries, England and China, which revealed as there was significant association between physical activity and socio-economic status of the family [6, 10, and 29]. The reason could be the difference in socio-cultural and economic development level of countries.

The limitation of this study is lack of generalizability due to it was conducted in three high schools of one region and adolescent age groups. It is also based on self-reported information which might have a recall bias as far as the timing is concerned. In conclusion the magnitude of physical activity is low. Regular physical activity was significantly affected by sex, age and access to gymnasium. Therefore, the local, regional and national administrators in collaboration with sport and health offices administrators have to better work towards making facilities (gymnasium and playground) accessible to the adolescents so as for the public. They have also better to work towards making all the facilities girls friendly and consider making safe and healthy environment along investment and developmental plan through availing enough spaces for leisure time physical activities.

## Supporting information

S1 File English Version Questionaire. (DOCX)

S2 File Amharic Version Questionare. (DOCX)

## Abbreviations

AOR: Adjusted Odds Ratio
DALYs: Disability Adjusted Life-Years
CVD: Cardio Vascular Disease
OR: Odds Ratio
NCD: None Communicable Disease
NHS: National Health Survey
PA: Physical Activity
HTN: Hypertension
WHO: World Health Organization
