# Supplementary material for: Self-reported physical activity status among adolescents in Debre Birhan town, Ethiopia: Cross-sectional study
Source: PLoS One. 2020 Feb 21;15(2):e0229522. doi: 10.1371/journal.pone.0229522 (PMC7034904; doi:10.1371/journal.pone.0229522)
Supplement: S1 File — (DOCX) [file pone.0229522.s001.docx]

CONSENT FORM

We are doing research on magnitude of physical activity, which is commonly recommended behavior to be healthy. We kindly invite you to be part of this research. Before you decide, you can talk to anyone you feel comfortable with about the research. We will not be collecting or retaining any information about your identity. Your decision will not result in any loss or benefits to which you are otherwise entitled. You have the right not to answer any single question, as well as to withdraw completely from the interview at any point during the process; additionally, you have the right to request the researcher not to use any of your responses. Your signature below indicates that you have decided to volunteer as a research participant for this study, and that you have read and understood the information provided above.

Subject's Signature: ___________________ Date _____________________

Investigator’s Signature: _______________________________

|  | **Part One: Sociodemographic Data** | |
| --- | --- | --- |
| 1  2 | What is your sex? Male 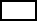 Female 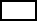  Age ___________ | |
| 3 | Grade 9 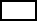 10 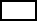 11 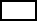 12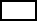 | |
| 4 | What is your religion: Orthodox Christian 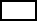 Muslim 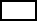  Protestant 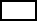 Other 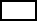 | |
| 5 | What is your family Monthly Income?______________________ | |
| 6 | Mother Education level  No education 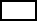  Elementary 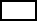  Secondary 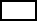  University college 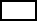 | 7. Father Education level  No education 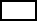  Elementary 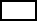  Secondary 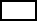  University college 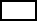 |
| 8 | Mother occupation  House wife 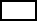  Private (merchant/farmer) 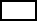  Government 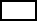  None government 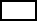  Other_________________ | 9. Father occupation  Farmer 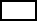  Private (merchant/ farmer) 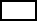  Government 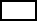  None government 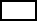  Other ____________ |
| 10 | Where do your families live? In the twon 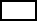 Tn rural area 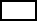 | |
| 11 | Which one of the following Items are available?  in your home? more than one options  Television 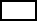  Satellite dish 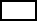  Computer 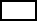  Internet service 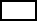 | 12. Residence type?  Private 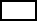  Rent 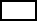  Condominium 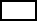 |
| 13  14  15 | Do you have personal cell phone? Yes 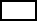 No 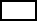  If your answer is yes, does your phone access internet service? Yes 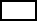 No 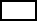  Do you use a social media? (e.g facebook, viber, telegram e t c ) Yes 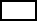 No 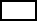 | |
| 16 | Is there a play ground near by your residence? Yes 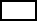 No 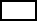 | |
| 17 | Is there sport gym nearby your residence? Yes 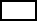 No 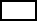 | |

| **Part Two: Perception Towards Physical Activity and Sedentary Behavior** |
| --- |
| 1. What do you feel about regular physical activity?   Very enjoyable 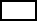 Enjoyable 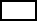 Not enjoyable 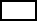   1. What do you think is the effect of regular physical activity on our health?   Good for health 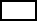 No benefit 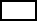 Bad for health 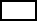   1. What do you think the effect of too much sitting on our health?   Bad for health 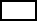 No harm for health 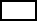 Good for health 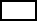 |

| **Part Three: Physical Activity** |
| --- |
| 1. During the **last 7 days** did you do **moderate** to **vigorous** physical activities like aerobics, running, weight lifting, marshal art, Gymnastic or fast bicycling **in your leisure time for at least 10 minutes**?   Yes No  If yes for how many days? ________________  For how long? _______________   1. In the last 7 days, did you play recreational games like football, volley ball, Basketball in your leisure time **for at least 10 minutes**?   Yes No  If yes for how many days? ________________  For how long? _______________ |
