# Supplementary material for: Self-reported physical activity status among adolescents in Debre Birhan town, Ethiopia: Cross-sectional study
Source: PLoS One. 2020 Feb 21;15(2):e0229522. doi: 10.1371/journal.pone.0229522 (PMC7034904; doi:10.1371/journal.pone.0229522)
Supplement: S2 File — (DOCX) [file pone.0229522.s002.docx]

AMHARIC VERSION

**መረጃ ለመስጠት ፈቃደኛ መሆንህን/ሽን ማረጋገጫ**

በአካል ብቃት እንቅስቃሴ ጥናት እየሰራን ስለሆነ በዚህ ጥናት ዉስጥ እንዲሳተፉ ጥያቄ ልናቀርብልዎት እንወዳለን፡፡ በጥናቱ ለመሳተፍ ከመወሰንዎ በፊት የሚያሳስብዎት ነገር ካለ ከዋናዉ ተመራማሪ እና ከፈለጉት አካል ጋር የመነጋገር ሙሉ ነጻነት አለዎት፡፡ በጥናቱ ስዓት ማንነትዎን የሚገልጽ ምንም አይነት መረጃ የማንዎስድ ሲሆን የሚሰጡት መረጃ ሙሉ በሙሉ በሚስጥር የሚያዝ ይሆናል፡፡ በዚህ ጥናት በመሳተፍዎ የሚደርስብዎት ምንም አይነት ጉዳት የሌለ ሲሆን የሚያገኙትም ቀጥተኛ ጥቅም አይኖርም፤ ከዛ ባሻገር ግን ችግሩ ያለበትን ደረጃ ለማወቅ የድርሻዎን ይወጣሉ፡፡ የፈለጉትን ጥያቄ ያለመመለስ የመለሱትን ጥያቄ ተመራማሪዉ እንዳይጠቀም የመጠየቅ መብትዎ የተጠበቀ ነዉ፡፡ ይህንን ተከትሎ ከታች የሚፈርሙት ፊርማ በዚህ ጥናት ለመሳትፍ ፈቃደኛ መሆንዎንና ከላይ የተሰጡትን መረጃወች አንብበዉ የተረዱ መሆኑን ያረጋግጣል፡፡

ፊርማ____________________________

ቀን____________________________

| **ክፍል አንድ፡- የግል መረጃ** | |
| --- | --- |
| 1. ጾታ**？** ወንድ 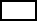 ሴት 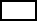 | |
| 1. እድሜ **？____________________** ዓመት | |
| 1. ክፍል**？** 9 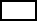 10 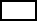 11 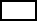 12 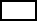 | |
| 1. ሐይማኖት**？**ኦርቶዶክስ 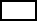 ሙስሊም 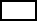   ፕሮቴስታንት 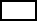 ሌላ**：**—————————— ይጥቀሱ | |
| 1. የቤተሰብህ ጠቅላላ የወር ገቢ**？** --------------------------------- ብር | |
| 1. የእናትህ/ሽ የትምርት ደረጃ**？**   ያልተማረች 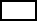  አንደኛ ደረጃ (1-8) 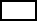  ሁለተኛ ደረጃ (9-12) 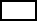  ኮሌጅ/ዩኒቨርሲቲ 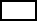 | 1. የአባትህ/ሽ የትምህርት ደረጃ**？**   ያልተማረ 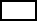  አንደኛ ደረጃ (1-8 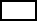  ሁለተኛ ደረጃ((9-12) 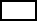  ኮሌጅ/ዩኒቨርሲቲ 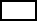 |
| 1. የእናትህ/ሽ ስራ**？**   የቤት እመቤት 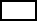  የግል/ንግድ 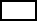  የመንግስት 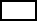  መንግሰታዊ ያልሆነ/NGO 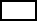  ሌላ ይጥቀሱ ----------------------------------- | 1. የአባትህ/ሽ ስራ**？**   ገበሬ 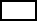  የግል/ንግድ 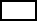  የመንግስት 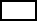  መንግሰታዊ ያልሆነ/NGO 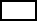  ሌላ ይጥቀሱ ----------------------------------- |
| 1. ቤተሰቦችህ/ሽ የሚኖሩት የት ነዉ**？** ከተማ 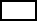 ገጠር 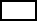 | |
| 1. የመኖሪያ ቤታችሁ ሁኔታ**？**   የግል ቤት 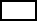 የኪራይ ቤት 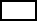 የጋራ ቤት 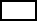 | |
| 1. ከሚከተሉት ዉስጥ በቤታችሁ ያለዉን እቃ ይምረጡ**？** ከአንድ በላይ መምረጥ ይቻላል   ቴሌቪዥን 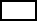 ዲሽ 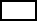  ኮሞፒዩተር 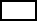 ኢንተርኔት 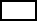 | |
| 1. የግል ሞባይል ስልክ አለህ/ሽ**？** አለኝ 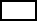 የለኝም 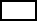 | |
| 1. ለጥያቄ ቁጥር 13 መልስህ/ሽ አለኝ ከሆነ ስልኩ ኢንተርኔት ይሰራል**？** ይሰራል 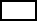 አይሰራም 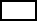 | |
| 1. የማህበራዊ ድረ ገጽ (face bool, viber, Telegram) ትጠቀማለህ**？** እጠቀማለሁ 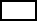 አልጠቀምም 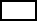 | |
| 1. በመኖሪያ አካባቢህ/ሽ የጨዋታ ሜዳ አለ**？** አለ 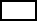 የለም 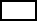 | |
| 1. በመኖሪያ አካባቢህ/ሽ የስፖርት ጂምናዚየም አለ**？** አለ 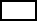 የለም 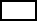 | |

| **ክፍል ሁለት፡- አተያይን በተመለከተ** |
| --- |
| 1. ዘወትር የሚደረግ የአካል ብቃት እንቅስቃሴ ምን አይነት ስሜት ይፈጥርብሀል**？**   በጣም ደስ የሚል 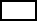 በመጠኑ ደስ የሚል 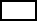 ደስ የማይል 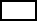   1. የዘወትር የአካል ብቃት እንቅስቃሴ ማድረግ በጤናችን ላይ ምን ያስከትላል**？**   ጤናማ ያደርገናል 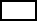 ምንም ጥቅም የለዉም 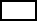 ጤናን ይጎዳል 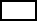   1. ብዙ ሰአት መቀመጥ በጤናችን ላይ ምን ያደርሳል ብለህ/ሽ ታስባለህ/ሽ**？**   ጤናን ይጎዳል 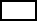 ምንም ጉዳት የለዉም 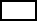 ጤናን ያሻሽላል 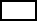 |

| **ክፍል ሶስት፡- የአካል ብቃት እንቅስቃሴ** |
| --- |
| 1. ባለፉት ሰባት ቀናት ከ 10 ደቂቃ በላይ ከመካከለኛ እስክ ከባድ የአካል ብቃት እንቅስቃሴ ማለትም እንደ ኤሮቢክስ ክብደት ማንሳት ሩጫ ማርሻል አርት እና የመሳሰሉትን ሰርተሃል/ሻል？   ሰርቻለሁ 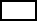 አልሰራሁም 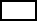 (መልስዎ አልሰራሁም ከሆነ ወደ ጥያቄ ቁጥር **2** ይለፉ)  1.1. ከሰራህ/ሽ ለስንት ቀን ____________________________________  1.2. ከሰራህ/ሽ በቀን ለምን ያህል ሰአት ________________________   1. ባለፉት ሰባት ቀናት ከ 10 ደቂቃ በላይ እንደ እግር ኳስ መረብ ኳስ ቅርጫት ኳስና የመሳሰሉትን አይነት ጨዋታዎች ተጫዉተሃል/ሻል？   ተጫዉቻለሁ 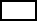 አልተጫወትኩም 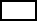 (መልስዎ አልተጫወትኩም ከሆነ ወደ ክፍል **አራት** ይለፉ)  ከተጫወትክ/ሽ ለስንት ቀን ______________________  በቀን ለስንት ሰአት ______________________  የተጫወትከዉ የትኛዉን የጨዋታ አይነት ነዉ  እግር ኳስ 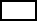  ቅርጫት ኳስ 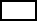  መረብ ኳስ 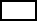  ሌላ ካለ ይጻፍ _________________________________________ |
